# Patient and Physician Perspectives on Pharmacotherapy in Parkinson’s Disease Psychosis: A Mixed-Methods Exploratory Study

**DOI:** 10.3390/pharmacy14010008

**Published:** 2026-01-13

**Authors:** Olaf Rose, Tobias Hinteregger, Eugen Trinka, Bernhard Iglseder, Johanna Pachmayr, Stephanie Clemens

**Affiliations:** 1 Institute of Pharmacy, Pharmaceutical Biology and Clinical Pharmacy, Paracelsus Medical University, Strubergasse 21, 5020 Salzburg, Austria; tobias.hinteregger@alumni.pmu.ac.at (T.H.); johanna.pachmayr@pmu.ac.at (J.P.); stephanie.clemens@pmu.ac.at (S.C.); 2 Center of Public Health and Health Services Research, Paracelsus Medical University, Strubergasse 21, 5020 Salzburg, Austria; 3 Department of Neurology, Centre for Cognitive Neuroscience, EpiCARE, Christian-Doppler University Hospital, Paracelsus Medical University, 5020 Salzburg, Austria; 4 Paracelsus Medical University Centre for Cognitive Neuroscience, Neuroscience Institute, Christian-Doppler University Hospital, 5020 Salzburg, Austria; 5 Karl Landsteiner Institute of Neurorehabilitation and Space Neurology, 5020 Salzburg, Austria; 6 Department of Geriatric Medicine, Christian Doppler Klinik, Paracelsus Medical University, Ignaz-Harrer-Straße 79, 5020 Salzburg, Austria

**Keywords:** Parkinson’s disease, Parkinson’s disease psychosis, patient, clozapine, quetiapine

## Abstract

Psychosis is a frequent and disabling non-motor complication of Parkinson’s disease (PD). Clozapine and quetiapine are widely used in the treatment of Parkinson’s disease psychosis (PDP). We conducted an exploratory study to compare patient experiences with physician prescribing practices. Patients with PDP hospitalized at a university center completed semi-structured interviews on perceived efficacy, adverse effects, and daily functioning. Neurologists and geriatricians attending training sessions completed a structured questionnaire on prescribing patterns, attitudes toward clozapine, and perceived treatment burden. Data were analyzed thematically and triangulated across cohorts. Eleven patients (mean age 81 years; nine treated with quetiapine, two with clozapine) were included. Most quetiapine-treated patients reported persistent hallucinations, sedation, dizziness, and reduced autonomy. Fourteen physicians completed the survey and most preferred quetiapine, citing monitoring logistics and agranulocytosis risk as barriers to clozapine. Overall, patient priorities centered on symptom control and independence, whereas physician decisions emphasized feasibility and safety. Facilitating clozapine monitoring and incorporating patient-reported outcomes into routine care may improve patient-centered PDP management.

## 1. Introduction

Parkinson’s disease (PD) is one of the most common neurodegenerative disorders, affecting millions of individuals worldwide [1]. Traditionally, PD has been understood primarily through its motor manifestations, such as tremor, rigidity, bradykinesia, and postural instability [2]. Yet, over the past decades, clinical and research attention has increasingly shifted to the recognition of non-motor symptoms, which often have an even greater impact on patients’ daily lives than motor disability alone [3]. Among these non-motor complications, psychosis has emerged as one of the most disabling and distressing syndromes [4,5]. Parkinson’s disease psychosis (PDP) is characterized by a spectrum of symptoms [6,7,8]. At one end are minor illusions, fleeting misperceptions, and the sense of a “presence” in the room. At the other end are vivid and recurring visual hallucinations, sometimes auditory hallucinations, and delusions that may center on themes of jealousy, paranoia, or misidentification [9]. PDP can occur in both early and advanced stages of Parkinson’s disease, although its frequency and clinical context differ across the disease course. Early manifestations often include minor hallucinations such as illusions, passage hallucinations, or a sense of presence, which may later progress to well-formed visual hallucinations and, less frequently, auditory or tactile hallucinations. Delusions, typically paranoid or misidentification-related, tend to emerge in more advanced stages and are associated with substantial functional impairment and caregiver burden.

The pathophysiology of PDP is multifactorial and incompletely understood, involving an imbalance between dopaminergic, serotonergic, and cholinergic neurotransmission within cortical and subcortical networks responsible for perception and reality monitoring [3,10]. In advanced disease stages, declining endogenous dopamine production in the substantia nigra necessitates increasingly complex dopaminergic treatment regimens. To prevent motor fluctuations, freezing, and OFF phases, higher dopaminergic stimulation is often accepted, frequently at the cost of dyskinesias. These high levels in dopamine D_2_ receptor stimulation not only contribute to motor complications but may also directly lead to PDP. Serotonergic mechanisms, particularly involving 5-HT_2_A receptors, further modulate vulnerability to psychosis and provide a neurobiological rationale for antipsychotic strategies that minimize dopaminergic blockade.

These experiences can be profoundly destabilizing, not only for patients themselves but also for their caregivers and environment. PDP can undermine trust, safety, and independence. As psychosis advances, caregivers frequently describe “losing” the person they knew, as hallucinations and delusions intrude on daily life and reshape relationships [11]. The onset of PDP often marks a turning point in the disease trajectory. Psychosis increases the risk of hospitalization, accelerates placement in nursing homes, and is associated with higher mortality [5,12]. It imposes significant emotional and physical burden on caregivers, who must cope not only with the practical challenges of mobility and dependence but also with the unpredictable and unsettling nature of psychotic episodes. For patients, psychosis is experienced as a loss of autonomy and dignity, stripping away their sense of reality and control.

Pharmacological treatment of PDP is uniquely challenging. Dopaminergic therapies with Levodopa, dopamine agonists or MAO-B inhibitors form the cornerstone of PD motor management. Unfortunately, they often exacerbate psychotic symptoms, leaving a narrow therapeutic window for motor improvements. Most antipsychotics, in turn, worsen motor function by blocking dopaminergic pathways [13]. Clozapine has consistently demonstrated robust efficacy in reducing hallucinations and delusions without exacerbating parkinsonian symptoms [14]. Clinical trials have shown clozapine to be highly effective at low doses in PDP, providing meaningful symptom relief while preserving motor control [15]. Its use, however, is limited by the risk of agranulocytosis, necessitating regular hematological monitoring. Although there is consensus to reduce the frequency of absolute neutrophil count monitoring over time, specific regulatory requirements continue to impose logistical and administrative barriers in many healthcare systems [16]. While these safeguards have reduced severe adverse events, they have created substantial logistical hurdles for prescribers and institutions. By contrast, quetiapine is widely prescribed despite the lack of consistent evidence for efficacy in PDP [17]. Its widespread use is largely driven by practical considerations, including the absence of mandatory monitoring and familiarity among prescribers.

Clozapine and quetiapine differ substantially in their mechanism of action and receptor binding profiles, which may contribute to their distinct clinical effects in Parkinson’s disease psychosis [18]. Clozapine exhibits relatively low affinity for dopamine D_2_ receptors and a higher affinity for serotonin receptors, particularly 5-HT_2_A, alongside activity at D_4_, muscarinic, histaminergic (H_1_), and adrenergic receptors. At the low doses typically used in PDP, this receptor profile allows antipsychotic effects with minimal interference with nigrostriatal dopaminergic transmission, thereby reducing the risk of motor symptom worsening. Quetiapine likewise shows low D_2_ receptor occupancy at clinically used doses, combined with antagonism at 5-HT_2_A, H_1_, and α_1_-adrenergic receptors. However, quetiapine’s comparatively strong antihistaminergic and adrenergic effects are associated with sedation and orthostatic symptoms, while its antipsychotic efficacy in PDP appears less consistent. Taken together, both agents share low D_2_ antagonism, but clozapine’s broader serotonergic and dopaminergic receptor interactions may contribute to antipsychotic efficacy at low doses, whereas quetiapine’s receptor profile may favor tolerability and sedation over robust control of psychotic symptoms. These pharmacodynamic differences are relevant when interpreting patient-reported outcomes and real-world treatment patterns in PDP. Clozapine has demonstrated antipsychotic efficacy at low doses that typically do not compromise motor function, reflecting a distinct pharmacodynamic profile compared with other antipsychotics [13,14,19]. Quetiapine, by contrast, is commonly used at doses that may induce sedation and tolerability effects without consistently achieving antipsychotic efficacy [13,14].

In this context, mandatory hematological monitoring for clozapine represents a component of pharmacological vigilance rather than solely an organizational constraint. These pharmacodynamic and dosing-related differences, together with monitoring requirements, directly influence drug use patterns and implementation in routine care, linking PDP treatment decisions to core questions in pharmaceutical and pharmacotherapy research. From a pharmacotherapeutic and translational standpoint, PDP highlights the complexity of implementing evidence-based drug therapies in real-world settings. Treatment decisions in PDP are shaped not only by clinical considerations but also by how pharmacological evidence is operationalized within regulatory frameworks, monitoring requirements, and healthcare delivery structures. Consequently, antipsychotic use in PDP can be viewed as an implementation-sensitive pharmacotherapy context, in which drug handling, monitoring pathways, and system compatibility may influence therapeutic uptake and continuity. From the perspective of pharmaceutical science and clinical pharmacy, PDP exemplifies the challenges of medication management in vulnerable populations. Beyond drug selection, pharmacotherapy in PDP involves structured dose titration, continuous benefit–risk evaluation, safety monitoring, and the translation of regulatory requirements into routine care processes. These elements are central domains of pharmacy practice and directly affect therapeutic accessibility, adherence, and continuity of care. In this setting, pharmacists operate at the interface between pharmacological evidence, system-level feasibility, and patient experience, including the assessment of treatment burden and patient-reported outcomes. Framing PDP within this pharmaceutical and implementation-oriented framework establishes its relevance for translational pharmacotherapy research, pharmacy practice, and pharmaceutical care.

From a pharmaceutical standpoint, patient-reported information extends the assessment of pharmacotherapy beyond clinical symptom ratings. Such information contributes to the evaluation of treatment efficacy, tolerability, and functional impact, which are central pharmaceutical outcomes in medication-related decision-making. Incorporating patient-reported information therefore supports a more comprehensive evaluation of pharmacotherapy in routine PDP care. In this context, patient-reported information can represent a later step in the pharmacotherapy continuum, complementing drug discovery and clinical efficacy trials by informing how medications perform under real-world conditions.

This mixed-methods study explored patient and physician perspectives on pharmacological treatment of PDP. It was not designed to assess effectiveness.

## 2. Materials and Methods

### 2.1. Study Design

This study followed an exploratory mixed-methods design integrating qualitative and quantitative data to examine pharmacological treatment of PDP. Mixed methods were chosen to capture both prescribing patterns and lived experiences of patients, allowing comparison between evidence-based recommendations, physician decision-making, and patient perspectives. The study comprised two parallel arms: semi-structured interviews with patients and a descriptive questionnaire survey of physicians. Integration of both strands was achieved through triangulation.

### 2.2. Setting

The study was conducted at the Departments of Neurology and Geriatric Medicine of the Christian-Doppler-Clinic, University Hospital of Paracelsus Medical University, Salzburg, Austria. Both departments provide specialized inpatient care for patients with advanced PD admitted for motor complications, non-motor symptoms, or psychosis.

### 2.3. Instrument Development

Questionnaires and interview guides were developed based on a focused literature review and expert discussion. Instrument development followed a two-step validation process including content and face validation [20,21]. Items addressed perceived efficacy of quetiapine and clozapine, experienced adverse effects, treatment-related burden for patients, caregivers, physicians, and healthcare institutions, and, for physicians only, antipsychotic choice and willingness to switch treatment. Content validation was performed by neurologists, pharmacists, and methodologists, followed by piloting with clinicians not included in the final sample. Minor revisions were made to improve clarity and structure.

### 2.4. Patient Survey

#### 2.4.1. Inclusion, Exclusion, and Recruitment

Eligible participants were patients with a confirmed diagnosis of PDP, who were receiving antipsychotic treatment, typically quetiapine or clozapine. Patients were required to be able to participate in a structured interview; caregiver or proxy support was permitted where appropriate. Exclusion criteria included psychosis unrelated to PD and inability to provide informed consent directly or via a legal representative. Patients were identified by attending neurologists and geriatricians during hospital admission. Participation was voluntary and did not affect clinical care. Written and oral study information was provided, and written informed consent was obtained. Participants could withdraw at any time without consequences.

#### 2.4.2. Data Collection

Data were collected using semi-structured face-to-face interviews combined with a structured questionnaire. Closed questions ensured comparability across participants, while open-ended questions captured individual experiences. Interview topics included psychotic symptoms, perceived treatment effects, adverse events, quality of life, caregiver burden, and attitudes toward treatment-related requirements such as blood monitoring. Interviews were conducted in the hospital setting, recorded verbatim, transcribed, and pseudonymized. Caregivers were invited to supplement responses when patients had difficulty expressing themselves.

#### 2.4.3. Data Handling and Analysis

Interview transcripts were anonymized and thematically analyzed. Coding was conducted iteratively to identify recurring patterns related to treatment efficacy, adverse effects, functional impact, and treatment trade-offs. Codes were grouped into broader themes such as sedation versus clarity, mobility and falls, caregiver burden, and acceptance of monitoring. Trustworthiness was enhanced through triangulation with physician survey data and team-based discussion of emerging themes.

### 2.5. Physician Questionnaire, Recruitment and Administration

Physicians were recruited during continuing education sessions and through ward-based contact at Salzburg University Hospital. Eligible participants were neurologists and geriatricians involved in the care of patients with PDP. Participation was anonymous and voluntary. The questionnaire was administered in paper form and digitized for analysis. No incentives were provided. The physician questionnaire was designed as an exploratory instrument with both structured and open-ended elements. While clozapine-associated agranulocytosis and hematological monitoring were explicitly included based on prior evidence indicating their relevance for prescribing decisions, respondents were encouraged to report additional adverse effects and considerations for both clozapine and quetiapine in free-text responses. This approach was chosen to capture physician-prioritized factors rather than to systematically assess the full adverse-effect profiles of the respective agents.

### 2.6. Analysis

Interviews were audio-recorded and transcribed using automated transcription software [22], followed by qualitative content analysis using MAXQDA according to Mayring’s approach [23]. Quantitative questionnaire data were analyzed descriptively using frequencies and proportions. Qualitative responses were coded thematically and integrated with patient data to identify concordance and divergence between perspectives.

### 2.7. Ethics, Data Protection, and Trustworthiness

This study was designed as an exploratory mixed-methods investigation and was not intended to generate statistically generalizable results. The limited sample size and single-center setting reflect the vulnerability of the study population and the focus on in-depth data collection rather than representativeness. Triangulation was applied at the level of perspectives rather than data types, integrating patient-reported experiences with physician-reported prescribing patterns to examine concordance and divergence in pharmacological treatment of PDP. Qualitative and quantitative components were analyzed separately and subsequently compared during interpretation to identify overlapping and contrasting themes relevant to pharmacotherapy decision-making. Participant selection followed a purposive approach. Patients were recruited based on a confirmed diagnosis of PDP and current antipsychotic treatment, aiming to capture lived experiences with commonly used pharmacotherapies. Physicians were included based on their active involvement in PDP treatment. This sampling strategy was chosen to support exploratory comparison across stakeholder groups rather than population-level inference.

Ethical approval was obtained from the Salzburg Ethics Committee (EC No. 1011/2024). All procedures complied with the Declaration of Helsinki and applicable data protection regulations. Written informed consent was obtained from all participants or their legal representatives. Data were anonymized, stored securely, and accessible only to the research team. Trustworthiness was supported through triangulation of data sources, reflexivity, and systematic documentation of analytic decisions.

To facilitate transparency and reproducibility, the full interview guides, physician questionnaire, coding framework, and analytic procedures are documented in detail and are available from the corresponding author upon reasonable request. The mixed-methods design, including sampling strategy, instrument development, triangulation approach, and analytic workflow, is described to allow replication in comparable clinical settings. Given the exploratory nature of the study, emphasis was placed on methodological transparency rather than statistical generalizability.

### 2.8. Use of Generative AI Tools

Generative artificial intelligence tools were used solely to support language editing, clarity, and stylistic refinement of the manuscript text. No generative AI tools were used for study design, data collection, data analysis, interpretation of results, or generation of figures or tables. All scientific content, analyses, and conclusions are the sole responsibility of the authors.

## 3. Results

### 3.1. Patient Characteristics and Clinical Profile

Over a period of two months, 172 patients diagnosed with PD were screened. Approximately 25 of these patients satisfied the inclusion criteria. Interviews were successfully conducted with eleven patients or, when applicable, their legal representatives. The remaining patients were unable to participate due to the progression of the disease and no available legal custodian. Of the patients included, nine were receiving quetiapine and two were receiving clozapine at the time of interview. Patients ranged in age from the late sixties to the mid-eighties (72–86 years, mean age 81.1 years). More female than male patients participated. Reported causes for PDP varied. Caregivers contributed additional observations when patients had difficulty expressing themselves. Demographics are presented in Table 1.

The prescribed antipsychotic medications for delusional symptoms in the surveyed patients included quetiapine (*n* = 9) and clozapine (*n* = 2). Quetiapine dosages varied from 12.5 mg to 125 mg per day. Specifically, quetiapine was prescribed in the following daily doses: 25 mg (five patients), 50 mg (two patients), 100 mg (one patient), and 125 mg (one patient). Clozapine was prescribed at 12.5 mg for both patients. Patients reported durations of antipsychotic therapy ranging from 1 week to 24 months. Psychotic episodes had been experienced for periods between 2 weeks and 18 years. Experienced symptoms included visual hallucinations (*n* = 10; 90.9%), acoustic hallucinations (*n* = 3; 27.3%), and tactile hallucinations (*n* = 1; 9.1%). More than one symptom could occur in the same patient.

### 3.2. Quantitative Results in Quetiapine-Treated Patients

**Figure 1:**
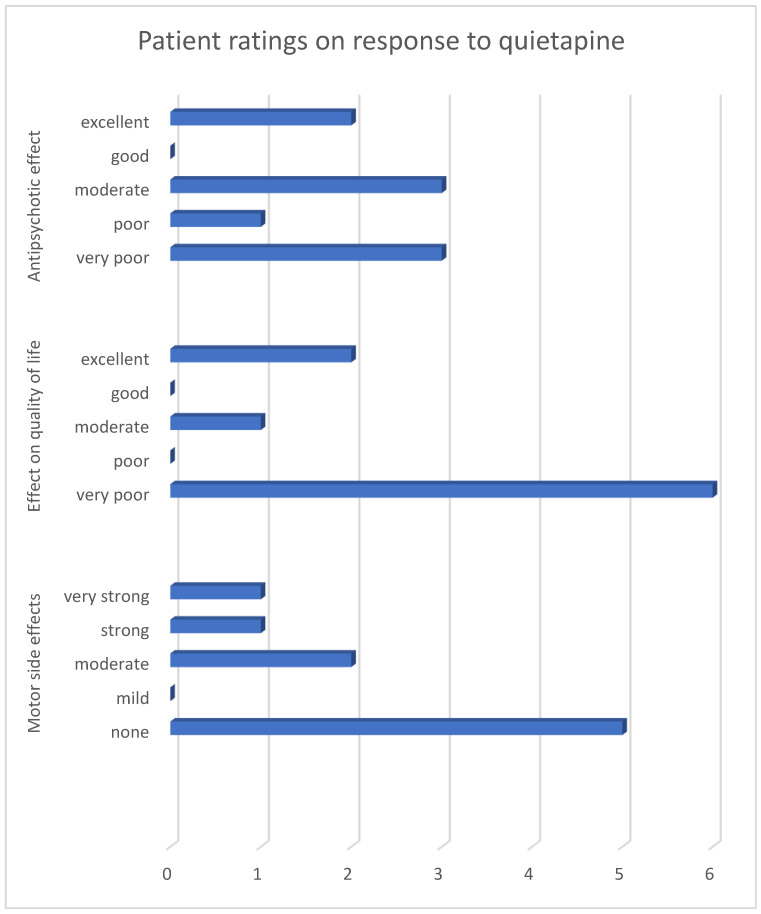
Reported effects of quetiapine on hallucinations, quality of life, and side effects among quetiapine-treated patients only. Numbers indicate the number of patients selecting each response option (*n* = 9). Due to the very small number of clozapine-treated patients (*n* = 2), quantitative visualization for clozapine was not performed.

Patient ratings demonstrated a heterogeneous response to quetiapine in the treatment of PDP (Figure 1). Overall, perceived antipsychotic efficacy was limited, with most patients indicating only partial or insufficient symptom control.

The impact on quality of life followed a similar pattern, with ratings predominantly reflecting a lack of meaningful improvement in daily functioning. In contrast, motor tolerability was generally rated more favorably, suggesting that adverse motor effects were not the primary driver of dissatisfaction with treatment.

Taken together, the quantitative results illustrate a divergence between tolerability and perceived therapeutic benefit, a pattern that becomes apparent when efficacy, quality of life, and motor side effects are considered jointly.

### 3.3. Qualitative Results

Patients described their PDP as vivid, intrusive, and often persistent perceptual experiences that interfered with daily activities and sense of reality. Hallucinations ranged from complex, person-related perceptions to threatening or bizarre visual phenomena and were frequently experienced as highly realistic, despite patients’ partial awareness of their unreal nature. Illustrative examples included the persistent perception of a non-existent person accompanying everyday activities:

“That’s my grandchild. He’s always with me in my flat, even though he’s not there.”

(Patient 1, pos. 17–19)

Other patients described disturbing visual hallucinations with threatening content:

“I see shadows. I see a demon running out of the sky.”

(Patient 2, pos. 11–18)

Most patients reported a lack of meaningful therapeutic effect under quetiapine. Descriptions frequently emphasized the absence of improvement in psychotic symptoms, with some patients perceiving symptom persistence or subjective worsening rather than stabilization. Dissatisfaction was commonly expressed in terms of unchanged daily experience rather than acute adverse effects.

Representative statements included the following:

“I don’t notice anything.”

(Patient 8, pos. 41)

“Well, not worse, but not better either. I’m not really satisfied with it.”

(Patient 5, pos. 26–27)

Most patients also reported a lack of meaningful therapeutic effect under quetiapine. Descriptions frequently emphasized the absence of improvement in psychotic symptoms, with some patients perceiving symptom persistence or subjective worsening rather than stabilization. Dissatisfaction was commonly expressed in terms of unchanged daily experience rather than acute adverse effects.

Representative statements included the following:

“I don’t notice anything.”

(Patient 8, pos. 41)

“It didn’t get better, not worse either. I’m not really satisfied with it.”

(Patient 5, pos. 26–27)

In contrast, a small number of patients reported a perceived positive effect of quetiapine on psychotic symptoms. These accounts described partial or noticeable improvement, although they were less frequently reported than statements indicating absent or insufficient treatment effects.

Illustrative statements included the following:

“I would say there are signs of improvement.”

(Patient 10, pos. 94)

“Yes, I would say it has improved significantly. It has improved a lot.”

(Patient 9, pos. 39)

Some patients reported adverse effects associated with quetiapine treatment, particularly dizziness, increased fall risk, and nausea. These experiences were described as affecting physical stability and daily functioning rather than acute or severe adverse reactions.

Representative statements included the following:

“Yes, falls have become more frequent” “Getting up in the morning became more difficult, because it was easy before”

(Patient 1, pos. 65–69)

“Cleary worse, clearly. And I feel nauseous or something like that”

(Patient 8, pos. 71)

Patients receiving quetiapine frequently reported persistence of hallucinations despite sedative effects. While treatment was sometimes described as calming, patients emphasized that sedation did not translate into meaningful changes in psychotic content or symptom resolution.

Representative statements included the following:

“No, that doesn’t work at all…not at all for me.”

(Patient 3, pos. 10)

“Nothing has really changed.”

(Patient 10, pos. 115)

Autonomy, quality of life, and social functioning emerged as central concerns in many patient interviews. Patients frequently described loss of independence and social withdrawal as burdensome consequences of their condition, while others emphasized the importance of effective symptom control for restoring stability in everyday life and family interactions.

Representative statements included the following:

“No, well, I just don’t feel needed anymore…lonely.”

(Patient 1, pos. 80)

“If that works, it would be a blessing, because then things at home might work out again.”

(Patient 11, pos. 146)

By contrast, both patients treated with clozapine reported marked and unambiguous improvement in psychotic symptoms. Their accounts differed qualitatively from those of quetiapine-treated patients in that symptom relief was described as clear, sustained, and directly perceptible in everyday life rather than as partial calming or sedation. Patients emphasized the disappearance of hallucinations and a return to a more stable and familiar daily experience.

One patient described the change as transformative:

“Great, I am so happy with it.”

(Patient 4, pos. 84)

Another emphasized the restoration of normality:

“They are all gone. I am very satisfied with it, great.”

(Patient 6, pos. 70–72)

### 3.4. Physician Prescribing Patterns

The survey of neurologists was conducted during an in-house training session, in which 14 participants completed the questionnaire. Physicians overwhelmingly preferred quetiapine to clozapine, despite acknowledging its limited efficacy. The primary reason cited for avoiding clozapine was the burden of monitoring. Most respondents identified mandatory blood tests and the risk of agranulocytosis as decisive factors against prescribing it.

**Figure 2:**
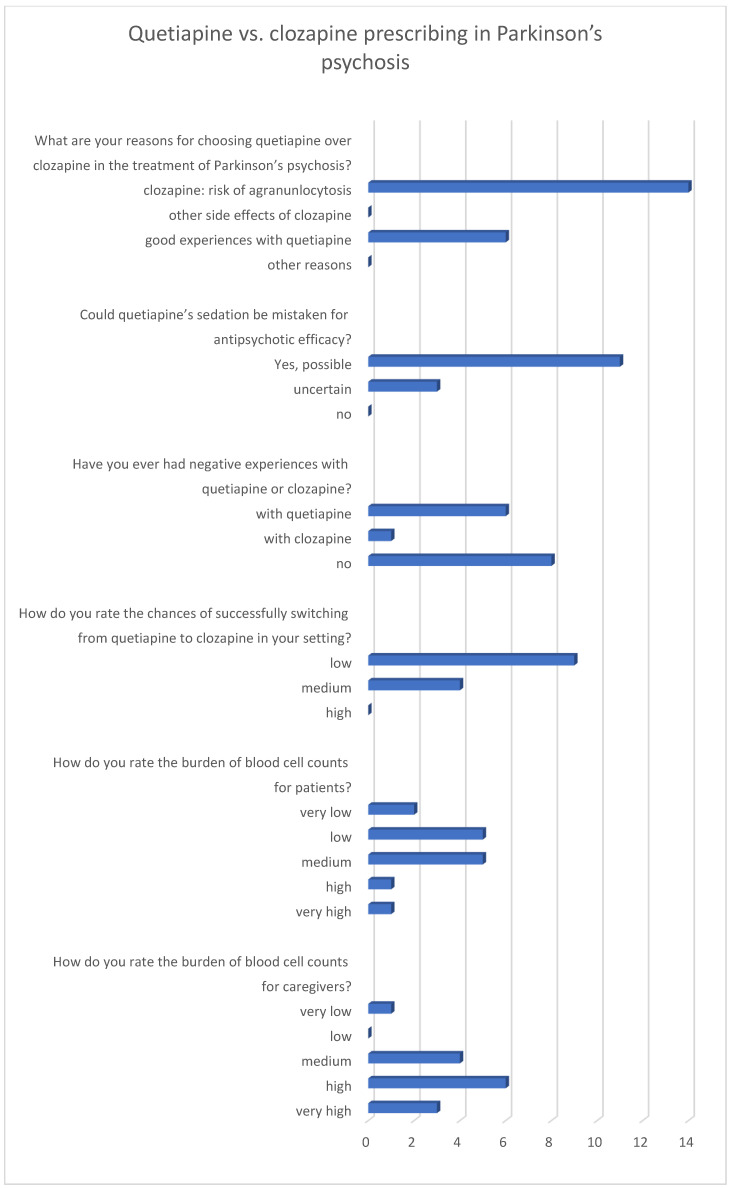
Neurologists’ ratings on antipsychotic therapy in Parkinson’s disease psychosis. Numbers represent the number of votes (*n* = 14).

When asked about quetiapine’s efficacy, most neurologists admitted that its sedative effect could easily be mistaken for antipsychotic benefit. Negative experiences were reported more frequently with quetiapine than with clozapine. Nevertheless, most neurologists regarded switching patients from quetiapine to clozapine as unlikely to succeed in their clinical context, largely due to limited confidence in the reliability of blood cell monitoring in the ambulatory setting. Finally, when asked about treatment burden, physicians rated it as low to medium for patients but high to very high for themselves and their institutions. This finding contrasted sharply with patient accounts, where quetiapine significantly reduced independence while clozapine restored it. Results are shown in Figure 2.

### 3.5. Integrative Summary

**Figure 3:**
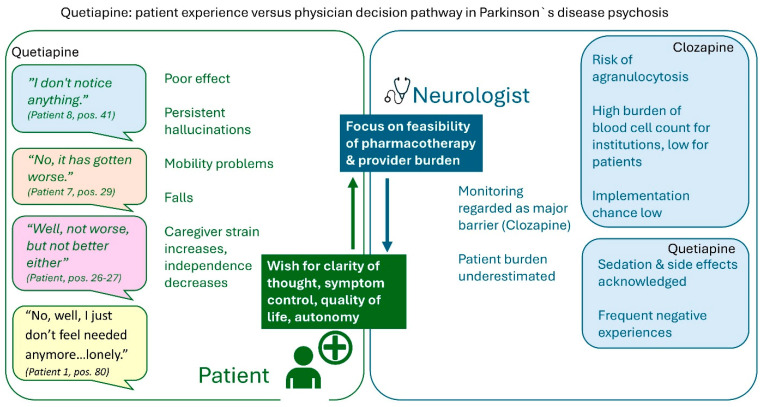
Mixed-methods matrix contrasting patient and neurologist perspectives on the management of Parkinson’s disease psychosis.

Integrated quantitative and qualitative data link patient-reported experiences with physician prescribing considerations in PDP. Patients reported limited perceived antipsychotic efficacy of quetiapine, with persistent psychotic symptoms, sedation, and minimal quality-of-life improvement despite acceptable motor tolerability. Physician data similarly indicated limited efficacy, while emphasizing feasibility, safety, and monitoring requirements. Two clozapine-treated patients reported subjectively meaningful symptom improvement; these observations are descriptive and non-generalizable. The integrated analysis juxtaposes patient experiences and physician decision pathways without inferential interpretation (Figure 3).

## 4. Discussion

This study provides a direct comparison between patient-reported experiences and physician prescribing behavior in the management of PDP. By centering patient narratives, the analysis highlights a clear divergence between perceived treatment effectiveness and routine clinical practice. Most patients described quetiapine as insufficient, reporting persistent hallucinations and limited functional benefit, whereas physicians predominantly favored quetiapine due to perceived logistical challenges associated with clozapine monitoring. This discrepancy underscores a misalignment between patient priorities and prescribing considerations. From a pharmaceutical perspective, these findings primarily reflect differences in medication use and implementation rather than comparative effectiveness between antipsychotic agents. Patient accounts demonstrated a clear ability to distinguish between sedation and genuine improvement of psychotic symptoms [24]. This observation challenges the assumption that sedation reflects therapeutic success [25] and reinforces the importance of patient-reported outcomes in evaluating treatment effectiveness, as previously demonstrated in schizophrenia research [26]. While only two patients in this study received clozapine, both reported substantial symptom relief and improved functioning. Given the very small number of clozapine-treated patients, these observations are descriptive in nature and must be interpreted cautiously. Nevertheless, they contrast with the frequent reports of impaired mobility, falls, nausea, and reduced independence under quetiapine. These observations should be interpreted cautiously but contrast with the frequent reports of impaired mobility, falls, nausea, and reduced independence under quetiapine. These findings emphasize the relevance of functional outcomes in PDP treatment. From the patient perspective, effective therapy is defined not by calming effects but by improvements in clarity, autonomy, and daily functioning. Persistent psychosis and treatment-related sedation were perceived as more burdensome than the practical inconveniences associated with monitoring requirements. When integrated with the quantitative findings, which showed limited perceived efficacy and quality-of-life benefit despite acceptable motor tolerability, a consistent pattern emerges across data sources.

Physicians acknowledged the superior efficacy of clozapine but emphasized organizational and safety-related barriers to its use, particularly the requirements for hematological monitoring. Despite recognizing that quetiapine’s sedative effects may be mistaken for antipsychotic efficacy, clinicians continued to prioritize feasibility and institutional constraints. Physicians generally perceived the treatment burden for patients as low to moderate, while rating the burden for themselves and healthcare institutions as high. Limited communicative capacity in advanced Parkinson’s disease may further contribute to underestimation of patient burden [27].

Beyond its exploratory findings, this study illustrates how patient-reported experiences can inform pharmaceutical care and medication management in complex neuropsychiatric conditions. While not intended to guide prescribing decisions, the results suggest that structured assessment of patient-reported outcomes may complement existing clinical evaluations and support more transparent discussion of treatment goals, trade-offs, and monitoring requirements in PDP. Such approaches may be applicable to other implementation-sensitive pharmacotherapies in vulnerable patient populations.

Taken together, these findings suggest that discrepancies between patient experience and prescribing practice in PDP are primarily driven by differing evaluative frameworks rather than by conflicting assessments of treatment effectiveness. The divergence raises important questions regarding how treatment burden and benefit are weighed in clinical decision-making. Structural barriers and system-level constraints appear to play a decisive role in shaping prescribing behavior, potentially at the expense of patient-centered outcomes. While concerns regarding safety and feasibility are legitimate, the findings suggest that current practice may insufficiently reflect patient priorities. In summary, the qualitative and quantitative results converge in highlighting an implementation-sensitive pharmacotherapy context rather than conflicting perspectives.

Overall, this exploratory study highlights the need for greater integration of patient-reported outcomes into PDP management and for structural solutions that facilitate access to effective treatments such as clozapine. Given the exploratory design, these findings should be interpreted as hypothesis-generating and as informing pharmaceutical and pharmacotherapy research rather than as guiding clinical recommendations. Aligning prescribing practices more closely with patient experience may help reduce the gap between evidence, clinical feasibility, and quality of life in PDP.

From a research perspective, the findings highlight several directions for future work. Larger, multi-center mixed-methods studies are needed to examine pharmacotherapy decision-making in PDP across different healthcare systems and monitoring infrastructures. In particular, future research should systematically assess how dosing strategies, treatment duration, comorbidities, and concomitant medications interact with patient-reported outcomes. In addition, implementation-focused studies evaluating alternative monitoring models for clozapine and structured integration of patient-reported outcomes into routine care may help bridge the gap between evidence-based treatments and real-world pharmacotherapy.

### Study Strengths and Limitations

The main strength of this study lies in the integration of patient and physician perspectives, allowing direct comparison between lived experience and prescribing rationale. This triangulation provides insights into both therapeutic outcomes and systemic influences on clinical decision-making. Limitations include the single-center design and small sample size, reflecting the challenges of recruiting patients with advanced PDP who are able to participate in interviews. The vulnerability and disease burden of this population constrained interview length and depth but underscore the clinical relevance of the findings. In addition, the very limited number of clozapine-treated patients precludes any conclusions regarding comparative effectiveness or broader applicability. Accordingly, observations related to clozapine should be understood as illustrative and not generalizable. Unmeasured comorbidities and concomitant medications may have influenced patient experiences and treatment perceptions. The variability in quetiapine dosing, and the wide range of treatment durations substantially limit the interpretability of the findings.

## 5. Conclusions

This exploratory mixed-methods study examined patient-reported experiences with antipsychotic treatment for PDP and their relationship to physician prescribing practices. By integrating qualitative patient narratives with quantitative patient ratings and physician-reported considerations, the study identified a recurring discrepancy between patient-evaluated treatment benefit and routine pharmacotherapy decision-making. These findings should be interpreted as descriptive and hypothesis-generating rather than as evidence for changes in clinical practice.

Across data sources, quetiapine was frequently prescribed and generally perceived as feasible from an organizational standpoint. However, patients often described limited perceived antipsychotic efficacy, persistence of hallucinations, and restricted functional benefit, despite sedative effects. Patient reports consistently differentiated between calming effects and meaningful improvement of psychotic symptoms, emphasizing clarity, autonomy, and daily functioning as key indicators of therapeutic success. These observations underscore the relevance of patient-reported outcomes for pharmacotherapy evaluation in PDP, particularly when treatment goals extend beyond symptom containment.

In contrast, the experiences reported by the small number of clozapine-treated patients in this study were characterized by marked symptom relief and perceived restoration of functional stability. Given the very limited number of patients receiving clozapine, these observations cannot be generalized and should be interpreted with particular caution. Rather than indicating comparative effectiveness, they serve to illustrate the type of treatment response described by individual patients under conditions where monitoring requirements were successfully implemented.

Physician responses highlighted that prescribing decisions in PDP are strongly shaped by feasibility considerations, safety concerns, and organizational constraints, particularly with regard to hematological monitoring. While clinicians acknowledged the potential for sedative effects to be misinterpreted as antipsychotic efficacy, system-level barriers and perceived implementation burden appeared to play a central role in shaping real-world pharmacotherapy. This divergence between patient-reported priorities and prescribing considerations suggests that pharmacotherapy decisions in PDP are influenced by differing evaluative frameworks rather than by conflicting assessments of treatment goals.

Overall, the findings of this study support the hypothesis that discrepancies between pharmacological evidence, patient experience, and prescribing practice in PDP are closely linked to implementation-related factors. Future research should examine these relationships in larger, multi-center studies and focus on implementation-sensitive pharmacotherapy, including monitoring models, medication management strategies, and systematic integration of patient-reported outcomes. Such work may help clarify how evidence-based treatments can be translated into routine care while aligning pharmacotherapy decisions with patient-defined outcomes and real-world feasibility.

## Figures and Tables

**Table 1:** Demographics of patients, participating in the survey and interview.

Characteristic	Category	*n*	%
Gender	Male	4	36.4
	Female	7	63.6
Age	72–76	2	18.2
	77–81	6	54.5
	82–86	3	27.3
Patient reported cause of psychosis (only one cause could be chosen)	Parkinson’s disease	2	18.1
	Parkinson’s disease-related medication	3	27.3
	Changes in medication	3	27.3
	Other/unknown	3	27.3

## Data Availability

The data presented in this study are available on request from the corresponding author due to privacy and ethical reasons.

## Abbreviations

The following abbreviations are used in this manuscript:

PD	Parkinson’s disease
PDP	Parkinson’s disease psychosis
ANC	Absolute neutrophil count
